# Three-dimensional visualization of intramuscular innervation in intact adult skeletal muscle by a modified iDISCO method

**DOI:** 10.1117/1.NPh.7.1.015003

**Published:** 2020-01-22

**Authors:** Yusha Li, Jianyi Xu, Jingtan Zhu, Tingting Yu, Dan Zhu

**Affiliations:** a Huazhong University of Science and Technology, Britton Chance Center for Biomedical Photonics, Wuhan National Laboratory for Optoelectronics, Wuhan, Hubei, China; b Huazhong University of Science and Technology, MoE Key Laboratory for Biomedical Photonics, Wuhan, Hubei, China

**Keywords:** skeletal muscle, whole-mount immunostaining, intramuscular innervation

## Abstract

Three-dimensional visualization of the innervation in skeletal muscles is helpful for understanding the morphological structure and function. iDISCO, a whole-mount immunolabeling and clearing technique, provides a valuable tool for volume imaging of intramuscular nerve fibers but suffers from the nonspecific staining caused by the anti-mouse secondary antibody when using the murine primary antibody. We developed a modified iDISCO method by introducing pretreatment of Sca*l*eCUBIC-1 reagent, termed m-iDISCO. The m-iDISCO method could eliminate the nonspecific staining and achieve uniform and complete labeling of nerve fibers in various muscles with mouse anti-neurofilament primary antibody. Combining the m-iDISCO method with light-sheet microscopy enabled us to visualize the innervation of adult mouse tibialis anterior and trace the nerve fibers from extramuscular branches to intramuscular terminal branches. This method represents an effective alternative for studying the innervation of intact skeletal muscles in health and disease.

## 1 Introduction

Three-dimensional (3-D) visualization of intramuscular innervation can be particularly valuable for understanding morphological structures and functions of the skeletal muscles.^1^ Immunostaining on sectioned tissue is a powerful approach for labeling molecules of interest and revealing cellular distribution in many biological research studies, but it often requires extensive labor and time for 3-D reconstruction with potential artifacts. To circumvent these issues, whole-mount immunostaining methods have been developed to label intact large tissues, but it is still quite challenging to achieve uniform and complete labeling due to the difficulty of antibody penetration.^2^^–^^6^

In recent years, tissue optical clearing technique has emerged to render tissue transparent for deep-tissue imaging.^7^^–^^11^ Some of the optical clearing methods can increase not only optical transparency but also macromolecule-permeability of tissues, which can facilitate antibody penetration to a certain extent. Various tissue optical clearing methods, such as 3DISCO, CUBIC, SWITCH, and CLARITY, have been combined with whole-mount immunostaining for labeling and imaging of large-volume tissues.^12^^–^^16^ iDISCO, a simple method with no need of customized setup, enables facile detection of immunolabeled structures throughout not only intact embryos but also dense adult tissues.^17^

The mouse anti-neurofilament (anti-NF) antibody produced by Developmental Studies Hybridoma Bank (DSHB, 2H3) is a widely used primary antibody for immunolabeling of intramuscular nerve fibers.^18^^,^^19^ And this antibody is also much more cost-effective than other anti-NF antibodies. But the anti-mouse secondary antibody used after this murine primary antibody can cause nonspecific staining on the sarcolemma and the fascia surrounding the muscle and can further hinder entrance of remaining secondary antibody into the muscle. The iDISCO method provides an important tool for volume imaging of intramuscular nerve fibers. However, it is unable to decrease the nonspecific staining caused by the anti-mouse secondary antibody.^17^

In this study, we aim to modify the iDISCO method to achieve 3-D visualization of the innervation of skeletal muscles with murine anti-NF antibody. First, we introduced a pretreatment method to modify the iDISCO method, termed m-iDISCO, applied it to label various adult mouse skeletal muscles with the mouse anti-NF antibody (2H3, DSHB), then compared with the iDISCO method. Finally, using the modified method combining with light-sheet fluorescence microscope (LSFM), we conducted 3-D visualization of the innervation of adult mouse tibialis anterior.

## 2 Materials and Methods

### 2.1 Animals

In this study, adult (two- to three-month-old) *Thy1*-GFP-M mice (Jackson Laboratory) were used. All experimental procedures were performed in strict accordance with the Experimental Animal Management Ordinance of Hubei Province, China, and were approved by the Institutional Animal Ethics Committee of Huazhong University of Science and Technology.

### 2.2 Sample Preparation

Adult mice were deeply anesthetized with an intraperitoneal injection consisting of a mixture of 2% chloral hydrate and 10% urethane (8  ml/kg) and then transcardially perfused with 0.01 M PBS (Sigma-Aldrich Co., St. Louis, Missouri) followed by 4% (w/v) paraformaldehyde (PFA, Sigma-Aldrich Co., St. Louis, Missouri) in 0.01 M PBS. The skeletal muscles were excised and postfixed overnight at 4°C in PFA, then the samples were washed with 0.01 M PBS at least three times.

For muscle slices, the intact muscles were embedded in 4% (w/v) agarose (Sigma-Aldrich Co., St. Louis, Missouri) and were cut with a vibratome (VT 1000s, Leica, Germany).

### 2.3 Antibodies

The antibodies used in this study included primary antibody: mouse anti-NF antibody (2H3, DSHB; dilution 1:100); secondary antibody: goat anti-mouse IgG Alexa Fluor 594 (115-585-146, Jackson ImmunoResearch Laboratories, Inc.; dilution 1:300).

### 2.4 m-iDISCO Protocol

**Fig. 1:**
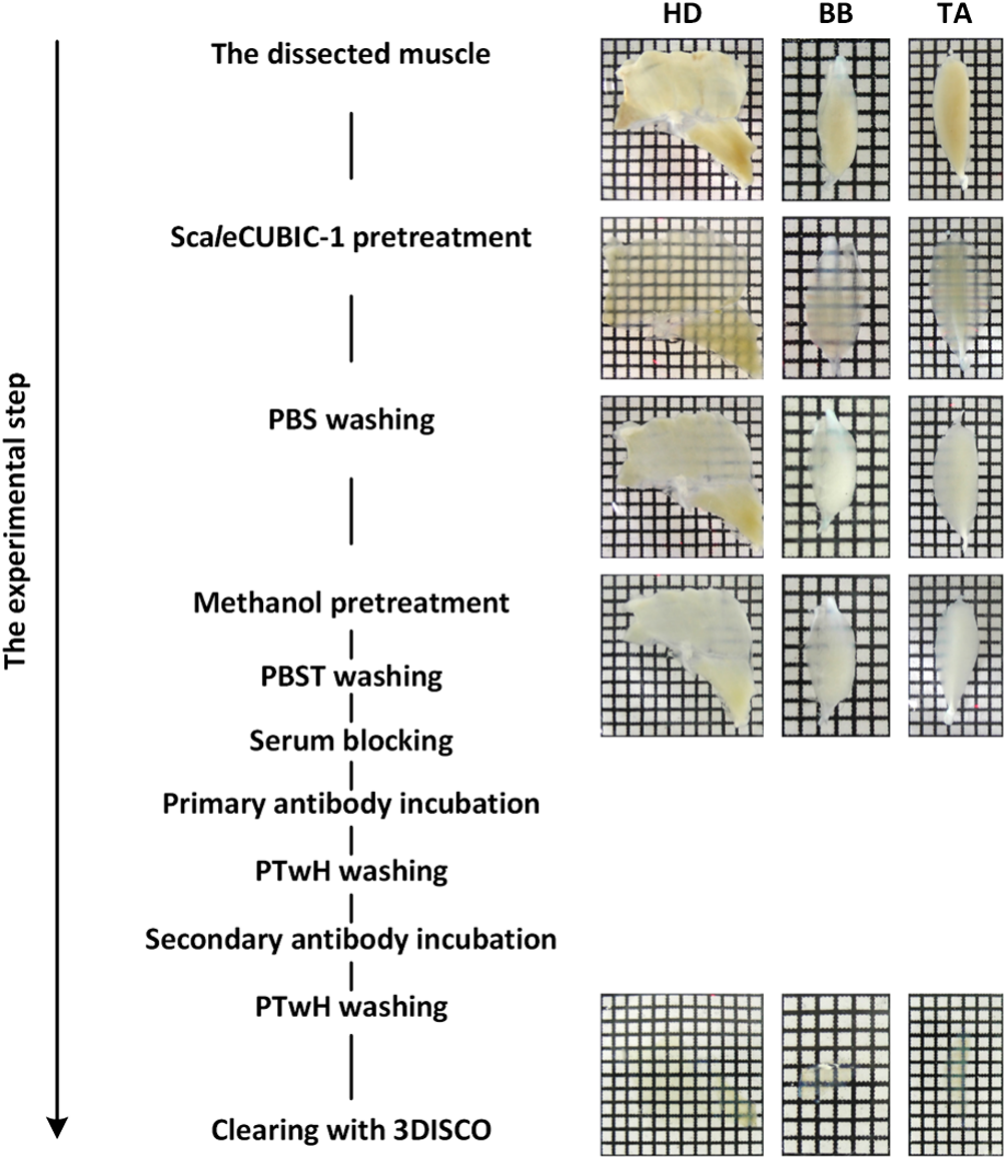
Overview of the m-iDISCO protocol and bright-field images of adult mouse skeletal muscles during the experimental steps. HD, half diaphragm; BB, biceps brachii; TA, tibialis anterior.

We developed the m-iDISCO method by introducing the pretreatment of Sca*l*eCUBIC-1 reagent to modify the iDISCO method. Overview of the m-iDISCO protocol is shown in Fig. 1.

First, Sca*l*eCUBIC-1 reagent was prepared as a mixture of 25 wt. % urea (Sinopharm Chemical Reagent Co., Ltd., China), 25 wt. % N,N,N′,N′-tetrakis(2-hydroxypropyl)ethylenediamine (Tokyo Chemical Industry Co., Ltd., Japan), and 15 wt. % Triton X-100 (Sinopharm Chemical Reagent Co., Ltd., China) according to the original literature of CUBIC.^12^ The fixed muscle samples were immersed in Sca*l*eCUBIC-1 reagent at 37°C with gentle shaking. The incubation time of Sca*l*eCUBIC-1 reagent depended on the size of muscles (Table 1). The pretreated muscles were washed thrice within 3 h with 0.01 M PBS at room temperature with gentle shaking. Then, the muscles pretreated with Sca*l*eCUBIC-1 reagent were performed methanol pretreatment, immunostaining, and tissue clearing by referring to the iDISCO protocol;^17^ the details are described below.

**Table 1:** The time of main processing steps for different skeletal muscles.

Muscle	Sca*l*eCUBIC-1 pretreatment	Serum blocking	Antibody incubation	Processing time^a^
Diaphragm	1 day	1 day	3 days	12 days
Biceps brachii	5 days	1 day	7 days	24 days
Tibialis anterior	7 days	1 day	7 days	26 days

a Processing time: from sample collection to imaging.

#### 2.4.1 Methanol pretreatment

First, samples were dehydrated in methanol (Sinopharm Chemical Reagent Co., Ltd, China) solutions at ascending concentration gradient (50 vol. %, 80 vol. %, 100%, and 100% in 0.01 M PBS) for 1  h/step. Samples were then bleached with 5% $H_{2}O_{2}$ in 20% dimethylsulfoxide/methanol (dimethylsulfoxide, DMSO, Sigma-Aldrich Co., St. Louis, Missouri; 30% $H_{2}O_{2}$:DMSO:methanol = 1:1:4 in volume) at 4°C overnight. After bleaching, samples were rehydrated in methanol solutions at descending concentration gradient (100% and 100%, 80 vol. %, 50 vol. % in 0.01M PBS) for 1  h/step. At last, samples were washed in 0.01M PBS twice within 2 h. Except bleaching, other steps were conducted at room temperature.

#### 2.4.2 Immunolabeling protocol

First, 0.2% Triton X-100 (Sigma-Aldrich Co., St. Louis, Missouri) in 0.01 M PBS (PBST) and 0.2% Tween-20 (Sigma-Aldrich Co., St. Louis, Missouri) in 0.01 M PBS with 10  mg/ml heparin (PTwH) were prepared. After methanol pretreatment, samples were incubated in PBST with 0.3 M glycine and 20% DMSO at 37°C overnight, then blocked in PTwH with 6% goat serum and 10% DMSO at 37°C for indicated time (Table 1). After blocking, samples were incubated in primary antibody diluted in PTwH with 3% goat serum and 5% DMSO for indicated time (Table 1). Then, samples were washed in PTwH for 1 day and incubated in secondary antibody diluted in PTwH with 3% goat serum and 5% DMSO for indicated time (Table 1). Samples were finally washed in PTwH for at least 1 day prior to clearing and imaging. Here, for the m-iDISCO and iDISCO methods, the antibody incubation and washing steps were conducted at room temperature.

#### 2.4.3 Tissue clearing

Immunolabeled tissues were dehydrated in tetrahydrofuran (Sinopharm Chemical Reagent Co., Ltd., China) solutions at ascending concentration gradient (50 vol. %, 70 vol. %, 80 vol. %, 100%, and 100% in ${\mathrm{dH}}_{2}O$). Finally, samples were incubated in dibenzyl ether (Shanghai Aladdin Bio-Chem Technology Co., Ltd., China) until cleared completely. The above experiments were carried out at room temperature. The diaphragms were dehydrated for 10 min/step while the biceps brachii and the tibialis anterior were dehydrated for 1  h/step.

### 2.5 Immunostaining of Muscle Slices

To explore the nonspecific staining caused anti-mouse secondary antibody, we stained 300-μm-thick muscle slices of the tibialis anterior only with the goat-anti-mouse antibody (115-585-146, Jackson ImmunoResearch).

First, muscle slices were blocked with 6% goat serum in 0.2% PBST for 1 h. Then, the blocked muscle slices were directly incubated with the goat-anti-mouse secondary antibody diluted in 0.2% PBST with 3% goat serum (dilution 1:500). Finally, muscle slices were washed with 0.2% PBST six times during 30 min.

### 2.6 Imaging

The diaphragms and muscle slices were imaged with an inverted confocal fluorescence microscope (LSM710, Zeiss, Germany) equipped with a 5×/0.25 dry objective (W.D. 12.5 mm), a 10×/0.5 dry objective (W.D. 2.0 mm), and a 20×/0.8 dry objective (W.D. 0.55 mm).

The biceps brachii and the tibialis anterior were imaged with an LSFM (Ultramicroscope, LaVisionBioTec, Germany) equipped with a 2×/0.5 objective and a macro zoom body (magnification steps from 0.63× to 6.3×).

When acquiring fluorescence images of samples stained with the iDISCO and m-iDISCO methods, respectively, we used the same acquisition settings.

### 2.7 Data Processing

The obtained images were analyzed with MATLAB, ImageJ, and Imaris software. MATLAB was used to calculate the signal and background intensity. ImageJ software was also used to perform maximum intensity projections (MIPs), image cropping and stitching, and analyze the grayscale values of pixels under marked line. Imaris software was used for 3-D and XZ-view reconstructions of z-stack images and tracing of nerve fibers within the muscles.

**Fig. 2:**
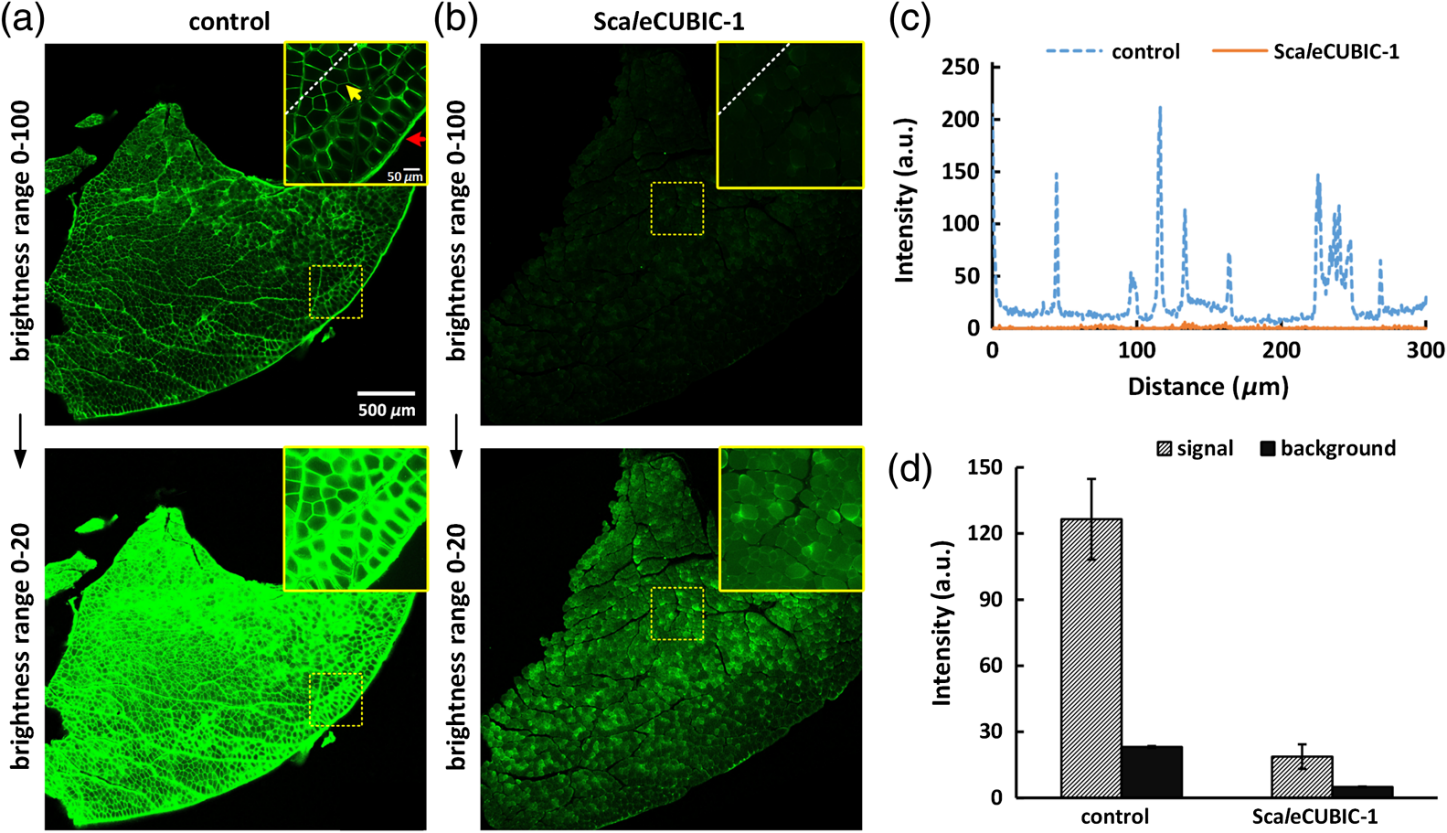
The nonspecific staining of the goat-anti-mouse secondary antibody on 300-μm-thick slices of adult mouse tibialis anterior. (a), (b) The 8-bit fluorescence images of muscle slices incubated with the secondary antibody. The muscle slices are from (a) fixed tibialis anterior and (b) one treated with Sca*l*eCUBIC-1 reagent for 7 days, respectively. The red and yellow arrows show the nonspecific binding in the fascia and sarcolemma, respectively. The upper and bottom rows in (a) and (b) are from the same data, respectively. The brightness ranges were adjusted to 0 to 20 (bottom row) to clearly show the outlines of muscle slices. (c) Normalized intensity profiles of the marked regions in (a) and (b) with white dashed lines. (d) Quantification of the fluorescence intensity in the signal and background areas of high-magnification images in the inner parts of muscle slices (n=3 for each condition).

For the quantification of the fluorescence intensity in Fig. 2(d), we first obtained the MIPs of high-magnification image stacks of muscle sections by ImageJ software. Then, the MIPs were separated into the signal and background areas by a threshold function in MATLAB software. Finally, the mean intensities of all pixels in the two areas were calculated and defined as the signal and background intensity.

For the segmentation of nerve fibers in Fig. 5, we first converted images into an 8-bit format by ImageJ software to facilitate image processing. Then, we opened 8-bit stack images with Imaris software and applied the “filament” tool for nerve segmentation. We added the “filament” and drew each nerve filament manually from starting point to end point in the autodepth mode. In addition, we added several “filaments” and selected different colors to differentiate nerve branches. Finally, we use the “snapshot” and “animation” tools to generated 3-D pictures and movies.

## 3 Results

### 3.1 Elimination of Nonspecific Staining by ScaleCUBIC-1 Pretreatment

As mentioned above, the mouse anti-NF primary antibody produced by DSHB (2H3) provides a cost-effective option for immunostaining of whole-mount skeletal muscles. However, the followed anti-mouse secondary antibody can abundantly bind to the dense sarcolemma and the fascia, especially for the ones with increased thickness in adult mouse skeletal muscles [Fig. 2(a)], which will substantially hinder penetration of antibodies and reduce the effective antibody binding. Although the iDISCO protocol can achieve whole-mount immunolabeling of various large tissues,^17^^,^^20^ it is unable to decrease the nonspecific binding when using this mouse anti-NF antibody. Sca*l*eCUBIC-1 reagent, originated from CUBIC protocol, shows high delipidation efficiency in mouse brain and kidney (close to 50%)^12^^,^^21^ and has been widely used for rapid lipid removal prior to clearing or embedding.^22^^,^^23^ Here, we investigated the influence of Sca*l*eCUBIC-1 processing of skeletal muscles on the nonspecific binding mentioned above [Fig. 2(b)]. We quantitatively compared the signal and background intensity without and with Sca*l*eCUBIC-1 pretreatment [Figs. 2(c) and 2(d)]. The results showed that the pretreatment of Sca*l*eCUBIC-1 reagent could weaken the binding between the anti-mouse secondary antibody with the sarcolemma and the fascia. Hence, we introduced the Sca*l*eCUBIC-1 pretreatment to modify the iDISCO protocol to label adult skeletal muscles and developed the m-iDISCO method for 3-D visualization of the innervation in intact skeletal muscles.

### 3.2 Whole-Mount Immunolabeling of Nerve Fibers in Adult Mouse Diaphragm

To validate the effectiveness of the m-iDISCO protocol, we first applied it to label the nerve fibers in adult mouse diaphragm, a relatively thin skeletal muscle, and compared with the iDISCO method.

**Fig. 3:**
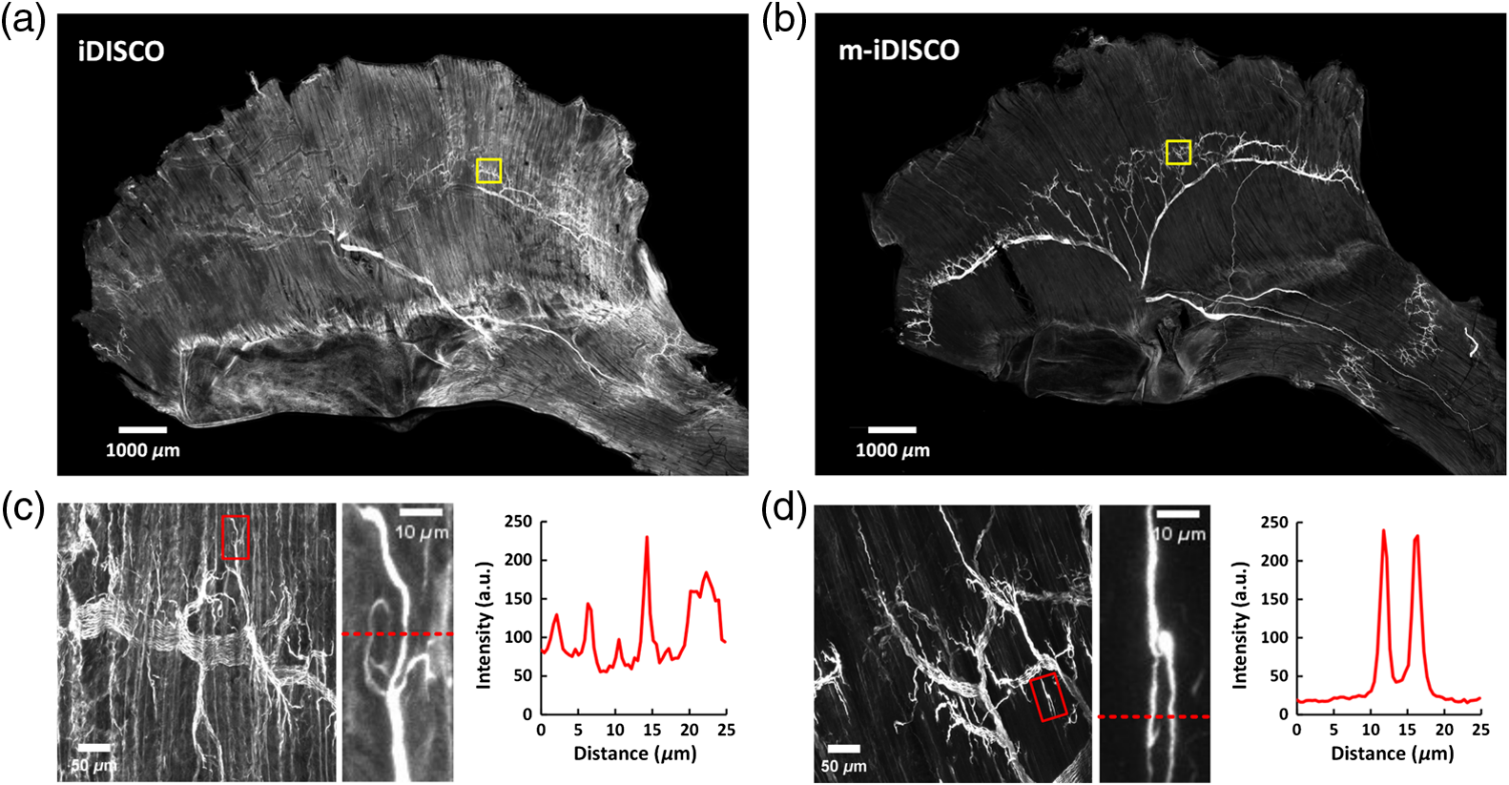
Whole-mount immunostaining with the mouse anti-NF primary antibody on nerve fibers in mouse diaphragm. (a), (b) The MIPs of images of the immunolabeled adult diaphragms by (a) the iDISCO and (b) m-iDISCO methods, respectively. (c), (d) The high-magnification images of the regions in (a) and (b) with yellow boxes, respectively. The locations of nerve endings were indicated with the red boxes. The plots are the gray values of pixels along the red dashed lines in the enlarged images.

By the iDISCO method, the distribution of nerve fibers in the diaphragm was not clear due to the high level of background signal [Fig. 3(a)]. Although we tried to prolong the time of serum blocking, it did not lower the background staining on the surface of the diaphragm (Fig. S1). In contrast, the m-iDISCO method performed well on the diaphragm and showed complete staining with decreased background staining and clearly distinguishable trunks and branches of nerve fibers [Fig. 3(b)]. We observed the nerve branches and the nerve endings by the high-magnification images and obtained the intensity plots at the nerve endings [Figs. 3(c) and 3(d)]. The relative signal intensity in areas of the nerve endings revealed that the muscle stained with m-iDISCO had lower background intensity and higher signal background ratio than the one stained with iDISCO. Hence, short-term Sca*l*eCUBIC-1 pretreatment (1 day) in the m-iDISCO method lowered the background staining but did not reduce the labeling of nerves.

Notably, the samples shown in Fig. 2 were only stained with the secondary antibody to investigate the nonspecific binding effect, whereas the samples shown in Fig. 3 were stained with the primary antibody followed by the secondary antibody to label the nerves. The sarcolemma and the fascia labeled were regarded as the signal in Fig. 2, whereas they were regarded as the background in Fig. 3. Both Figs. 2 and 3 demonstrated that the labeling on the sarcolemma and the fascia could be reduced after Sca*l*eCUBIC-1 pretreatment.

### 3.3 Whole-Mount Immunolabeling of Nerve Fibers in Adult Mouse Biceps Brachii and Tibialis Anterior

Furthermore, we compared the iDISCO method with the m-iDISCO method for labeling adult thick skeletal muscles. Here, we chose two typical muscles, including the biceps brachii from forelimb and the tibialis anterior from hind limb. After labeling, the muscles were imaged with LSFM and reconstructed with commercial software.

**Fig. 4:**
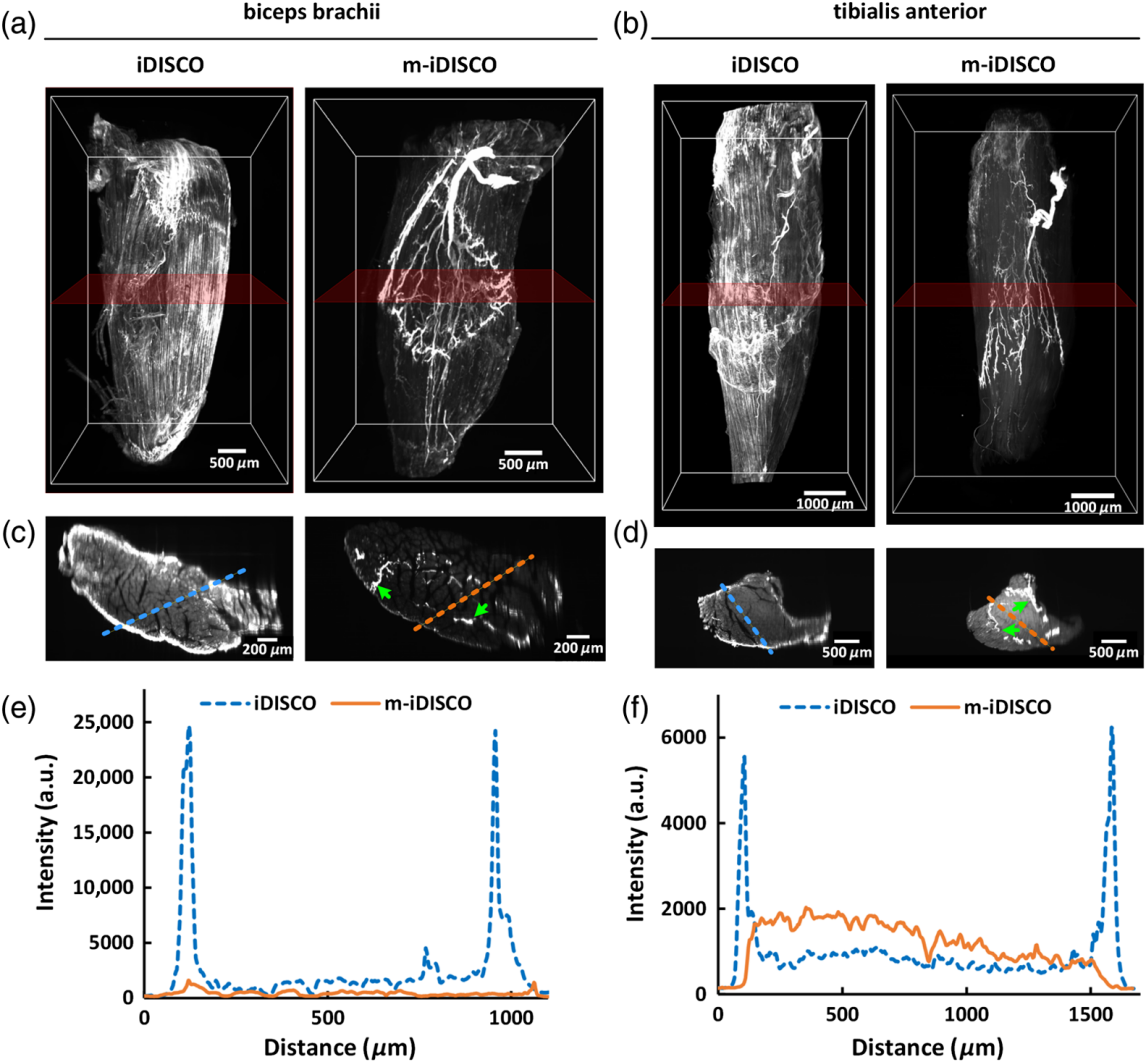
Whole-mount immunostaining with the mouse anti-NF primary antibody on nerve fibers in adult mouse biceps brachii and tibialis anterior. (a), (b) 3-D reconstruction of the biceps brachii and the tibialis anterior, immunostained with the iDISCO and m-iDISCO methods, respectively (Video 1). (c), (d) The 150-μm-thick MIPs of the images in XZ direction. The rough locations are marked in (a) and (b) with translucent red frames, and the green arrows indicate the labeled nerve fibers. (e), (f) Normalized intensity profiles of the background signal at the surface and the inside of the muscles. The locations are marked in (c) and (d) with the dashed lines. (Video 1, MPEG, 11.6 MB [URL: https://doi.org/10.1117/1.NPh.7.1.015003.1]).

The 3-D reconstruction of the muscles stained with the iDISCO method showed that there were some nerve fibers detected on the bone surface of the muscles, but no detection inside of the muscles in the 3-D volumes [Figs. 4(a) and 4(b), Fig. S2(a)]. While for m-iDISCO-stained muscles, the distributions of nerve fibers in the inner part of the muscles were clearly presented [Figs. 4(a) and 4(b), Video 1]. We reconstructed XZ-view of the image stacks, obtained the 150-μm-thick MIPs of images in the middle of the muscles, and quantitatively measured the background intensity at the surface and the inside of the muscles [Figs. 4(c)–4(f); Fig. S2(b)]. The results showed that, for iDISCO-stained muscles, the background intensity was higher on the surface than the inside of the muscles and the nerve fibers could not be distinguishable. In contrast, when stained with m-iDISCO, the background intensity was uniform for the entire muscles and the nerve fibers were labeled uniformly as indicated with the green arrows in Figs. 4(c) and 4(d).

The nonspecific binding between the anti-mouse secondary antibody and the muscles caused the background signal. As shown in Figs. 4(c)–4(f), the secondary antibody richly binded the fascia and the sarcolemma outside of the muscle, which made it difficult to penetrate into the inside when use the iDISCO method. While with the m-iDISCO method, the antibodies could effectively penetrate into the muscles to label the nerves inside owing to the pretreatment of Sca*l*eCUBIC-1. Hence, Sca*l*eCUBIC-1 pretreatment could not only weaken the nonspecific binding caused by the anti-mouse secondary antibody but also increase the penetration of the antibodies into the muscles for effective labeling. Notably, Figs. 4(e) and 4(f) showed that for the biceps brachii, m-iDISCO staining produced a relatively lower background signal inside the tissue than iDISCO, whereas for the tibialis anterior, m-iDISCO staining produced a relatively higher background signal inside the tissue than iDISCO. We speculated that the biceps brachii got a fuller pretreatment than the tibialis anterior due to its smaller size, which induced a slightly lower background signal with less binding between the sarcolemma inside the muscle with the anti-mouse secondary antibody.

### 3.4 3-D Visualization of the Innervation in Adult Mouse Tibialis Anterior

**Fig. 5:**
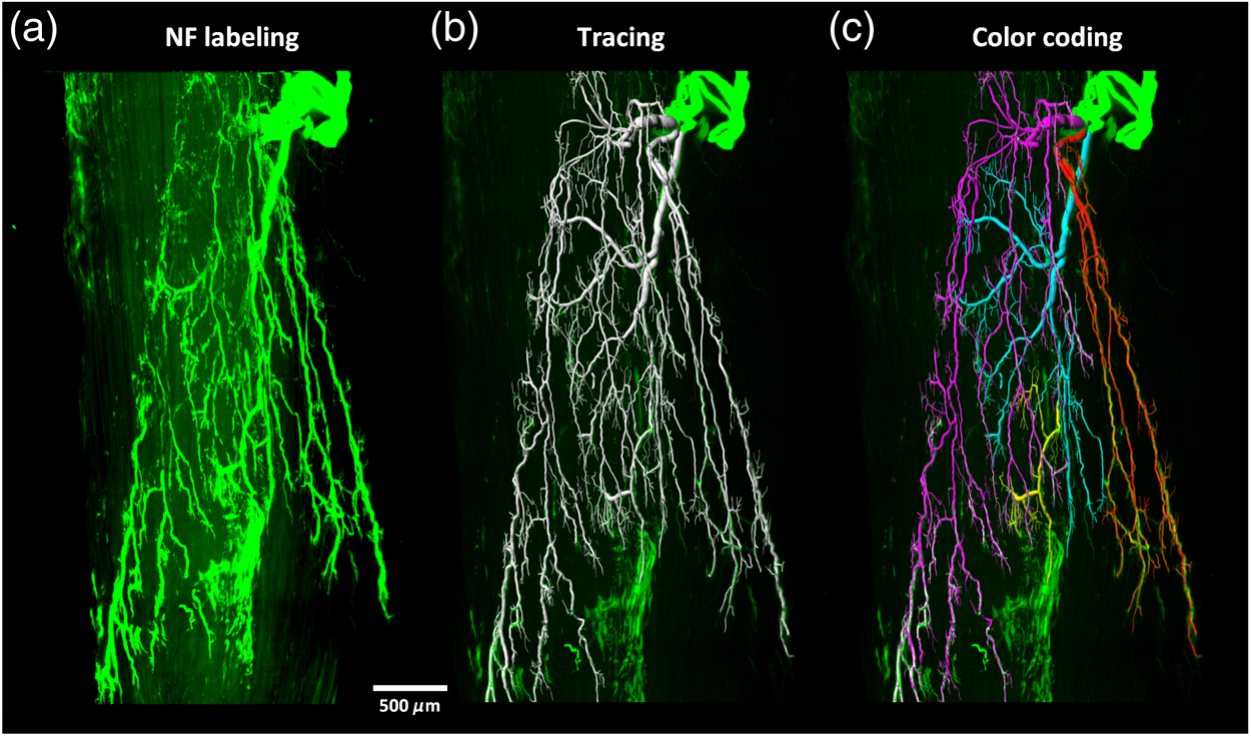
3-D visualization of the motor nerve fibers in adult mouse tibialis anterior (Video 2). The tibialis anterior was immunolabeled and cleared with the m-iDISCO method, then imaged with LSFM. The images were segmented and the nerve branches were coded with different colors. (a) LSFM images, (b) segmentation, and (c) color coding of different branches. NF, neurofilament. (Video 2, MPEG, 11.2 MB [URL: https://doi.org/10.1117/1.NPh.7.1.015003.2]).

After m-iDISCO staining of adult tibialis anterior, we obtained the 3-D distribution of intramuscular innervation and traced the motor nerve fibers from extramuscular branches to intramuscular terminal branches (Fig. 5; Video 2). As known, the tibialis anterior is innervated by the deep peroneal nerve, which enters into the tibialis anterior from the posterior side.^24^^,^^25^ The 3-D reconstruction and tracing of the nerve branches demonstrated that the motor nerve fibers in the tibialis anterior were distributed in an organized pattern of lamella, as shown with different colors in Fig. 5.

## 4 Discussion

Here, we developed a modified iDISCO method for 3-D visualization of intramuscular innervation in intact adult skeletal muscle, which we have called m-iDISCO. The m-iDISCO method achieved whole-mount anti-NF immunolabeling and clearing of intact adult mouse skeletal muscles, including diaphragm, biceps brachii, and tibialis anterior, and allowed 3-D analysis of intramuscular nerve fibers.

Notably, most studies focused on thin and small muscles of young mice in previous research due to the difficulty of labeling or imaging large muscles.^18^^,^^26^ Adult muscles are necessary objects when studying the muscle development and pathology.^27^ But the thickness of fascia surrounding the muscle increased and the tissues become denser with age, the antibody penetration into muscles of adult mice is more difficult than that of young mice. The modified protocol described in this work achieved whole-mount labeling of adult tibialis anterior (about 3-mm thick), which is thicker than muscles such as extensor digitorum longus^28^ and lumbricales.^29^ It is supposed that the pretreatment of Sca*l*eCUBIC-1 reagent in the m-iDISCO method might eliminate hindrance of fascia and could facilitate antibody penetration.

In this study, we used a mouse anti-NF primary antibody to label the nerve fibers in skeletal muscles. The anti-mouse secondary antibody could bind to the sarcolemma and the fascia surrounding the mouse muscles. This rich nonspecific binding caused the strong background staining outside of the muscles, also hindered the entrance of the secondary antibody into muscles, leading to incomplete labeling. As described in above results, Sca*l*eCUBIC-1 reagent could weaken this nonspecific labeling on skeletal muscles. A possible reason is the utilization of high-level detergent (15%), Triton X-100, in Sca*l*eCUBIC-1 reagent, which can maximize lipid removal and break down the cell membrane.^7^ However, for the same reason, Sca*l*eCUBIC-1 pretreatment also risks lowering epitope concentrations and potentially weakens immunostaining. So the influence of Sca*l*eCUBIC-1 pretreatment on the antigen–antibody reaction still needs to be verified before performing the m-iDISCO method with other antibodies.

In general, each experiment for screening appropriate antibodies requires extensive labor, time, and cost. This work demonstrated the labeling results with mouse anti-NF primary antibody (2H3, DSHB) by the m-iDISCO method, and these results can provide valuable references for the researchers. In addition, the mouse anti-NF primary (2H3, DSHB) used in this study is relatively cheaper (∼$0.4 for a muscle, $40 for 1 ml) than some other anti-NF primary antibodies (e.g., AB5539, Millipore, ∼$30 for a muscle, $689 for 50  μl). It provides a great and cost-effective option to study the innervation of various skeletal muscles in health and disease combined with the m-iDISCO method.

In summary, we have modified the iDISCO method by introducing the Sca*l*eCUBIC-1 reagent for pretreatment. We obtained 3-D distribution of intramuscular nerve fibers in various skeletal muscles and conducted 3-D tracing of the innervation of adult mouse tibialis anterior. This method potentially provides an option for 3-D histological analysis of various skeletal muscles, facilitating to understand structural–functional relationship of skeletal muscle in physiological and pathological condition.
