# Supplementary material for: Three-dimensional visualization of intramuscular innervation in intact adult skeletal muscle by a modified iDISCO method
Source: Neurophotonics. 2020 Jan 22;7(1):015003. doi: 10.1117/1.NPh.7.1.015003 (PMC6977403; doi:10.1117/1.NPh.7.1.015003)
Supplement: Supplementary file 1 [file NPh_007_015003_SD001.doc]

**Three-dimensional visualization of intramuscular innervation in intact adult skeletal muscle**

**by a modified iDISCO method**

**Yusha Li^a,b^, Jianyi Xu^a,b^, Jingtan Zhu^a,b^, Tingting Yu^a,b,*^, Dan Zhu^a,b^**

^a^Huazhong University of Science and Technology, Britton Chance Center for Biomedical Photonics, Wuhan National Laboratory for Optoelectronics, Wuhan, Hubei, China

^b^Huazhong University of Science and Technology, MoE Key Laboratory for Biomedical Photonics, Wuhan, Hubei, China

*yutingting@hust.edu.cn

**Supplementary Materials**

**
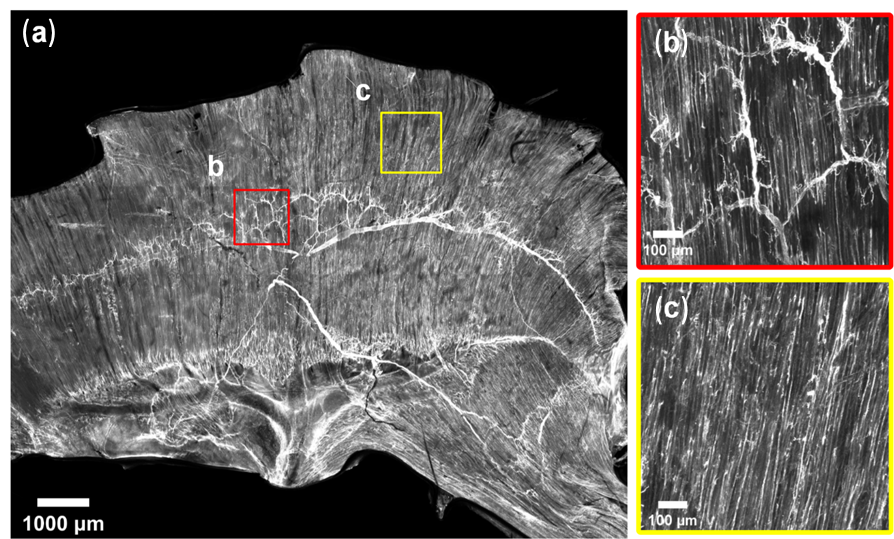
**

**Fig. S1** The staining effect by prolonging the time of serum blocking, when immunostained with mouse anti-NF primary antibody (2H3, DSHB). The adult mouse diaphragm was immunolabeled with the iDISCO method, thereinto the samples was blocked in 6% goat serum for 3 days. (a) The MIPs of images of the immunolabeled adult diaphragm. (b), (c) The high-magnification images of the regions in (a) indicated with red and yellow boxes. The red (b) and yellow (c) boxes show the signal area and the background area, respectively.


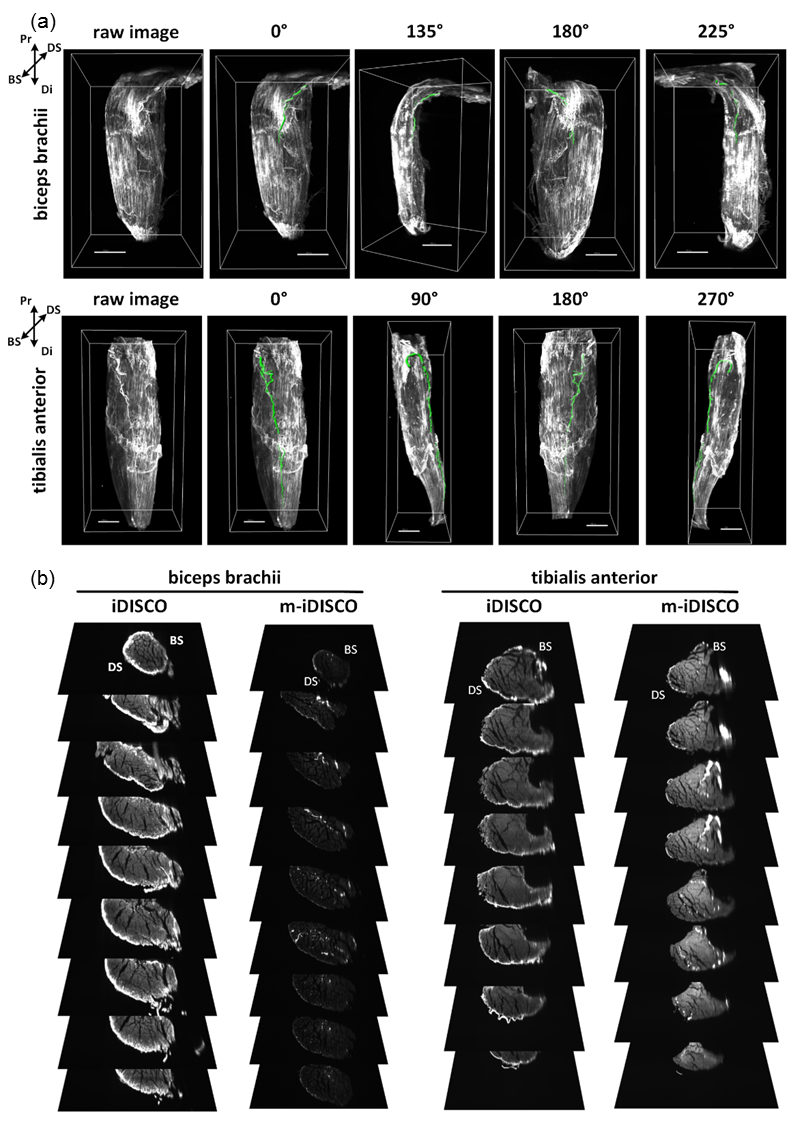


**Fig. S2** Reconstruction of the immunostained biceps brachii and tibialis anterior. (a) 3D reconstruction of the muscles immunostained with the iDISCO method. We rotated the muscles along the vertical axis to show the location of the segmented nerve fibers (green), and found that only the nerves on the bone surface of the muscles were labeled. Scale bar, 1000 μm. BS, bone surface; Di, distal end; DS, dorsal surface; Pr, proximal end. The orientation coordinates are for the raw images. (b) Resampling cross-sections of the intact muscles. Each image is the 20-μm-thick MIP with a 400-μm interval for the biceps brachii and the 40-μm-thick MIP with an 800-μm interval for the tibialis anterior.
